# MicroRNA-145 Increases the Apoptosis of Activated Hepatic Stellate Cells Induced by TRAIL through NF-κB Signaling Pathway

**DOI:** 10.3389/fphar.2017.00980

**Published:** 2018-01-12

**Authors:** Junfa Yang, Qingxue Liu, Shiyang Cao, Tao Xu, Xiaofeng Li, Dandan Zhou, Linxin Pan, Changyao Li, Cheng Huang, Xiaoming Meng, Lei Zhang, Xiao Wang

**Affiliations:** ^1^School of Pharmacy, Anhui Medical University, Hefei, China; ^2^Anhui Province Key Laboratory of Major Autoimmune Diseases, Anhui Institute of Innovative Drugs, Hefei, China; ^3^The Key Laboratory of Anti-inflammatory and Immune Medicines, Ministry of Education, Anhui Medical University, Hefei, China; ^4^School of Life Sciences, Anhui Medical University, Hefei, China; ^5^Department of Radiology, The First Affiliated Hospital of Anhui Medical University, Hefei, China

**Keywords:** liver fibrosis, miR-145, hepatic stellate cells, TRAIL, apoptosis, ZEB2, NF-κB signaling

## Abstract

During the liver fibrosis recovery stage tumor necrosis factor-related apoptosis-inducing ligand (TRAIL) can effectively induce apoptosis of activated hepatic stellate cells (HSCs). Normal hepatic stellate cells are resistant to TRAIL cytotoxicity. Therefore, enhancing the sensitivity of TRAIL-induced apoptosis of HSCs may be useful to treat hepatic fibrogenesis. Here, we demonstrated that miR-145 and TRAIL were down-regulated in both liver fibrosis tissue samples and transforming growth factor-β1 induced HSCs, concomitant with increased the expression of ZEB2. In addition, we found that mimics-mediated over-expression of miR-145 led to resistance to the ZEB2 expression and up-regulation of the TRAIL-induced apoptosis after treatment of LX-2 cells with TRAIL. Furthermore, ZEB2-siRNA transfected LX-2 cells showed the increased sensitivity to TRAIL-induced apoptosis. Whereas, opposite results were obtained in miR-145-inhibitor group or ZEB2 plasmid group. Moreover, miR-145 regulated ZEB2 gene expression by specifically interacting with the 3′-UTR of ZEB2 mRNA to inhibit the expression of ZEB2. Further studies showed that the over-expression of ZEB2 could inhibit TRAIL-induced apoptosis via inhibiting nuclear factor-κB (NF-κB) signaling pathway in LX-2 cells. Collectively, our data suggest that up-regulation of miR-145 can down-regulate ZEB2 expression, consequently promoting TRAIL-induced apoptosis in LX-2 cells through NF-κB signaling pathway, which facilitates the resolution of liver fibrosis.

## Introduction

Liver fibrosis is the common results of chronic liver injury of any etiology. It is characterized by an increase in extracellular matrix (ECM) components (Sato et al., 2003; Fagone et al., 2016). For many years, advanced fibrosis and cirrhosis are considered to be irreversible conditions even after removal of the injurious agent. Moreover, the fibrosis and cirrhosis of liver represent major cause of morbidity and mortality worldwide. However, current evidence indicated that liver fibrosis is reversible as liver injury subsides (Fallowfield, 2011; Lee and Friedman, 2011). Spontaneous recovery from liver fibrosis includes degradation of ECM proteins, apoptosis of activated hepatic stellate cells (HSCs) and regeneration of hepatocytes (Degos et al., 2011; Peverill et al., 2014). Key to this process is the discovery that reversion of fibrosis is accompanied with clearance of activated HSCs by apoptosis. Therefore, modulation of apoptosis of activated HSCs could be an important complementary pathway in the pathogenesis of fibrosis.

Tumor necrosis factor (TNF)-related apoptosis-inducing ligand (TRAIL), belongs to the TNF super-family, have the ability to induce cellular apoptosis through caspase-3 activation mechanism but not in most normal cells including hepatocytes (Holoch and Griffith, 2009; Xu et al., 2016). Recent studies have shown that the selective induction of HSCs apoptosis by TRAIL has been proposed as an important mechanism of terminating and potentially reversing liver fibrosis. Although the underling mechanisms regulating the apoptotic pathways of TRAIL-induced HSCs have not been fully elucidated, microRNAs (miRNAs) are excellent candidates to provide epigenetic orchestration of this process (Volkmann et al., 2007; Tang et al., 2009). In this study, we revaluated this issue in more detail and showed that miRNAs were involved in the TRAIL-induced apoptosis of activated HSCs.

The miRNAs, which play a key role in biological processes (Nedaeinia et al., 2017), are a class of endogenous small non-coding RNAs of size 19–25 nucleotides. Notably, recent evidence has revealed that the activation, proliferation, and apoptosis of activated HSCs were regulated by miRNAs (He et al., 2012; Noetel et al., 2012; Vettori et al., 2012). Importantly, miR-145 is considered as a regulator in some fibrosis diseases. Our previous study indicated that miR-145 plays a role in limiting the development of liver fibrosis by markedly blocking the activation and proliferation of HSCs (Zhou et al., 2016).

ZEB2, belongs to the zinc-finger E-box binding proteins family (Verschueren et al., 1999), can be positively mediated by transforming growth factor-β1 (TGF-β1) signaling (Comijn et al., 2001). The expression of ZEB2 was increased in radiation-induced pulmonary fibrosis have been identified in previous reports, which suggested a possible function of ZEB2 in modulating fibrosis diseases (Balli et al., 2013; Song et al., 2013). Additionally, many researchers have pointed out that HSCs activation can be inhibited by the nuclear factor-κB (NF-κB) signaling pathway, which plays an important role in apoptosis of HSC-T6 (Ding et al., 2011; Kong et al., 2013; Chen et al., 2014). One recent study reported that a decrease in ZEB2 expression resulted in reduced high affinity IgE receptor (Fc𝜀RI)-mediated activation of NF-κB signaling in mast cells (Barbu et al., 2012). Although these studies imply that ZEB2 has an effect on liver fibrosis, the regulation effect of ZEB2 to TRAIL-induced apoptosis of activated HSCs during through NF-κB liver fibrosis remains unclear.

In the current study, we found that miR-145 expression is highly decreased in liver fibrosis tissues and activated HSCs, and that up-regulation of miR-145 exacerbated TRAIL-induced apoptosis of activated HSCs. Mechanistic studies suggest that miR-145 regulated the apoptosis of activated HSCs induced by TRAIL by targeting ZEB2 through NF-κB signaling pathway.

## Materials and Methods

### Animal Models of Liver Fibrosis

Male mice weighting between 16 and 20 g were all purchased from Center for Experimental Animal of Anhui Medical University (Hefei, Anhui, China). The animal experiments and protocols were authorized by the institutional and local committee on the care. The mice were randomly grouped into normal groups and experimental groups. The experimental group mice were administrated with biweekly intraperitoneal injection of 20% solution of CCl4 (Shantou Xilong Chemical Plant, China) in peanut oil for 6 weeks while the normal groups were administered with the same volume of peanut oil only for 6 weeks. Mice were killed after 6 weeks. Livers and serum were collected for future analysis.

All experiments were performed according to the institutional ethical guidelines for laboratory animal care and use of Anhui Medical University. Procedures involve animals and their cares were conducted in conformity with NIH guidelines and was approved by Animal Care and Use Committee (number: LLSC20150348).

### Histopathology

For routine histology, the liver tissues were preserved in 10% formalin (Solarbio, Beijing, China) and embedded in paraffin and cut into 50-μm thick sections, which were prepared and stained with hematoxylin and eosin (H&E) and Masson staining by using standard methods. All images of pathological changes were captured with the Olympus BX-145 microscope (Olympus, Japan).

### Measurement of Alanine Aminotransferases (ALT) and Aspartate Aminotransferases (AST) Analysis

In separated serum samples, alanine aminotransferase (ALT) and aspartate aminotransferase (AST) activities from each serum sample were assessed by using commercial kits (jianchenbio, Nanjing, China) according to manufacturer’s direction with an auto analyzer.

### Primary HSCs Isolation

Primary HSCs were extracted via pronase/collagenase perfusion and differential centrifugation. The mice were treated with hydrolyzed trichloroacetaldehyde (Aladdin, American). After placing a line around the portal vein, a special needle was inserted into the vein. Then perfusion buffer perfused liver via portal vein. After the liver swelled, the cannula in the inferior vena cava was opened to allow draining. Subsequently, the liver was perfused in turn with enzymes fix after the blood was washed away and then the liver tends to white. Finally, the liver was gently removed from the abdominal cavity and placed in a sterile dish. Then the livers were disrupted to prepare cells suspension which was filtered to remove residual debris. After cell suspension centrifugation by several times, the cell suspension density was adjusted with Nycodenz mixture. The primary HSCs were acquired from the gradient centrifugation.

### Cell Culture

HSC-T6 cells were purchased from Cell Bank of Chinese Academy of Sciences (Shanghai, China). HSC-T6 cells were cultured in Dulbecco’s modified Eagle’s medium (DMEM, Hyclone) with 10% fetal bovine serum (FBS, sijiqing, Hangzhou, Zhejiang, China). LX-2, human immortalized HSC line, was acquired from Dr. S. L. Friedman (Mount Sinai School of Medicine) and cultured in DMEM containing 10% FBS (Gibco, United States). Cell cultures were maintained at 37°C in a humidified atmosphere containing 5% CO_2_. When grown to 80–90% confluence, cells were inoculated into six-well plates for culture and incubated for 12 h before transfection.

### Flow Cytometry

LX-2 cells were seeded in the six-well plates and incubated overnight, and then were treated with indicated regents for 48 h. The treated cells were harvested by using centrifugation at 1800 rpm after washed twice in PBS. After the cells were resuspended with 400 μl of annexin, the apoptosis of cells were detected by annexin V-FITC–propidium iodide (PI) double staining, using Annexin V-FITC Apoptosis Detection Kit II (BD Biosciences) according to the protocol. Experimental analysis was performed on flow cytometer (Beckman Coulter). All experiments were repeated three times.

### Double Immunofluorescence Staining

The liver fibrosis tissues were used for double immunofluorescence. Then tissues were permeabilized in 0.2% Triton X-100 including 1% BSA for 10 min, and blocked in 10% BSA for 1 h. To investigate the colocalization of ZEB2 and α-SMA, the tissues were treated with FITC-conjugated anti-α-SMA antibody (1:50 dilution, Boster, China) following Cy3-conjugated anti-ZEB2 antibody (1:50 dilution). All images were captured with confocal immunofluorescence microscopy (Olympus, Tokyo, Japan). The red represents ZEB2 and α-SMA as green fluorescence.

### Transfections and Dual-Luciferase Assay

Cells were seeded into a 96-well plate and co-transfected with miR-145 mimics and PYr-MirTarget-ZEB2 3′-UTR plasmid, and then assessed for luciferase reporter activity at 24 h post-transfection. Luciferase activity assay were performed in accordance with the manufacturer’s protocols in a dual-luciferase reporter assay for the sequential measurement of firefly and Renilla Luciferase Assay System (Promega). Cotransfection of a Renilla luciferase plasmid (pRL-TK) was performed to normalize the assays.

### miRNA Mimics, miRNA Inhibitor, and siRNA Transfection

To down-regulate or up-regulate the expression of miR-145 and ZEB2, miR-145 mimics, inhibitor, ZEB2 small interfering (si) RNA or corresponding control RNA were used. The transfection was performed by using lipofectamine^TM^ 2000 (Invitrogen, United States) in accordance with the manufacture’s protocols. Transfection after 6 h, the culture medium was changed and TGF-β1 was added. LX-2 cells were cultured at 37°C at an atmosphere of 5% CO_2_ in a humidified incubator for 24 h and collected for real-time PCR or Western blot analysis. miR-145 mimics, miR-145 inhibitor, and ZEB2 siRNA were purchased from GenePharma (Shanghai, China). The oligonucleotides sequences were listed:

miR-145 inhibitor: 5′-AGGGAUUCCUGGGAAAACUG GAC-3′,
miR-145 mimics: 5′-GUCCAGUUUUCCCAGGAAUCCCUG GAUUCCUGGGAAAACUGGACUU-3′.
ZEB2-siRNA (rat): 5′-CCAUCUCUGCUCAGAGUCCAA TT-3′,
5′-UUGGACUCUGAGCAGCAGAGCAGAUGGTT-3′; and Negative control: 5′-UUCUCCGAACGUGUCACGUTT-3′, 5′-ACGUGACACGUUCGGAGAATT-3′.

### Plasmid Transfection Analysis

Over-expression plasmid of ZEB2 (ZEB2-OE) was obtained by amplifying complementary DNA (cDNA) coding for ZEB2 cDNA and inserting cDNA coding for ZEB2 into destination vectors by Gateway cloning (Invitrogen, United States). The primers used were listed as following:

ZEB2-F: 5′-GGGGTACCCCATGAAGCAGCCGATCAT-3′,
ZEB2-R: 5′-GCTCTAGAGCTCACATGCCATCTTCC-3′.

The N-terminal region of ZEB2 coding region containing the predicted CARD domain was cloned into pEGFP-C2 vector by using restriction sites *Xba*I and *Bam*HI. Cell transfection was carried out with the Lipofectamine^TM^ 2000 (Invitrogen, United States) in the light of the manufacturer’s manuals.

### Quantitative RT-PCR Analysis

RNA was isolated from liver fibrosis tissues or the HSCs by using Trizol Reagent (Invitrogen) according to the manufacturer’s instructions. Total RNA quality and density were assessed by using the Thermo Scientific NanoDrop 2000 Spectrophotometer (Thermo Fisher Scientific, United States). To qualify miRNA or mRNA, RNA was reverse transcribed to cDNA by using transcriptor first-strand cDNA synthesis kit (Bio-Rad, United States) following the manufacturer’s protocols. After that, real-time PCR was carried out in a detection system with SYBR-Green Master Mix (Bio-Rad). Real-time PCR primers were acquired from Invitrogen. For the miR-145 expression analysis, the one-step miRNA real-time PCR Detection Kit (biomics, Nantong, Jiangsu, China) was used according to the manufacturer’s protocols. U6 small nuclear was used as endogenous control. All of measurements were performed three times and GAPDH was used as an endogenous control for mRNA expression. The following primers of genes were used:

ZEB2 (human) (forward: 5-CCCTTCTGCGACATAAATA CGA-3;
reverse: 5-TGTGATTCATGTGCTGCGAGT-3),
GAPDH (human) (forward: 5-ACCACAGTCCATGCCAT CAC-3;
reverse: 5-TCCACCACCCTGTTGCTGTA-3).

### Protein Isolation and Western Blot Analysis

Liver tissues and HSCs were lysed in RIPA buffer for protein extraction by using centrifugal separation. Then we collected upper supernatant. After extraction, protein concentration was estimated by a BCA protein assay kit (Boster, China). Proteins extracted were loaded on to 10% sodium dodecyl sulfate-polyacrylamide gel electrophoresis and electroblotted onto nitrocellulose membranes (Millipore, Bedford, MA, United States). After blocked with 5% non-fat milk in TBST, the PVDF membranes were washed with TBST buffer at least for three times and cultured with special primary antibodies at 4°C overnight. Then the PVDF membranes were washed for three times with TBST buffer. Next, the membranes were incubated with corresponding secondary antibody for 1 h. Immunoreactive bands were detected with ECL-chemiluminescent kit (ECL-plus, Thermo Fisher Scientific). Image J software was used to quantify and analyze intensities of the bands. β-Actin was developed as a loading control to verify the equal loading of proteins. Primary antibodies were as follows: ZEB2 (Santa Cruz Biotechnology, United States); TRAIL, Col. I, Caspase-3 (Bioss, Beijing, China), α-SMA (Sigma, United States), β-actin (Bioworld, United States).

### Statistical Analysis

Data was illustrated with values presented as the means ± SD from at least three separate experiments. The differences among groups were determined by using an unpaired *t*-test or a one-way ANOVA. Results were considered to be statistically significant at values of *p* < 0.05. All results were analyzed by using SPSS 17.0 software.

## Results

### Establishment of the Liver Fibrosis Models

**FIGURE 1:**
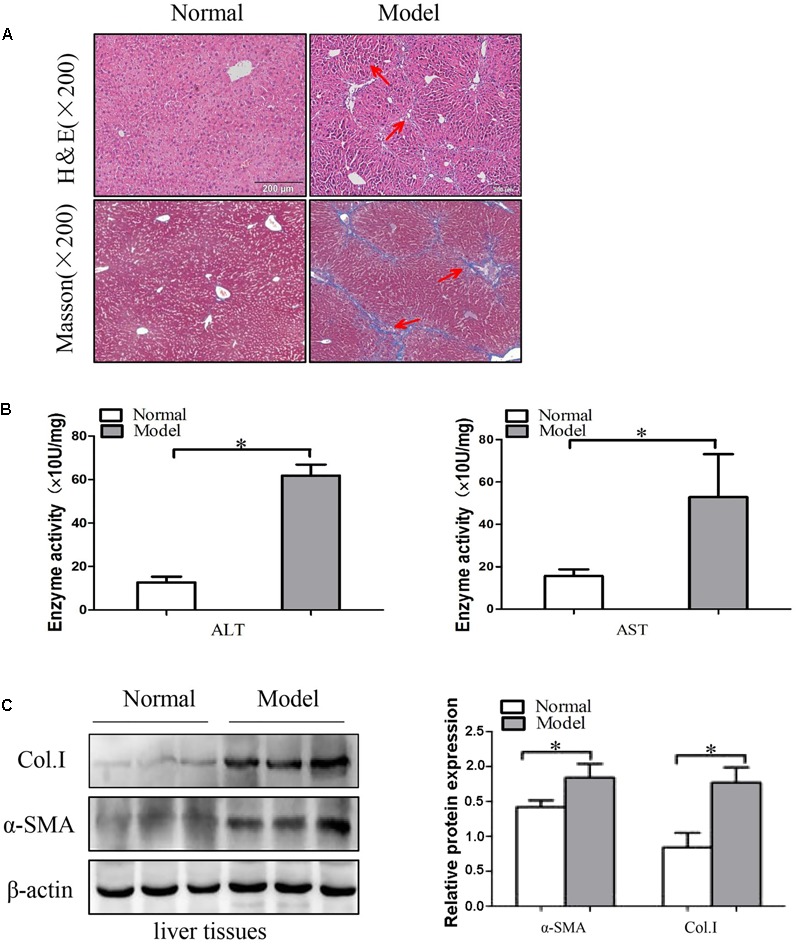
The expression of α-SMA and Col. I during liver fibrosis. **(A)** Pathological observations of mice liver fibrotic tissues sections and vehicle normal sections, stained with hematoxylin and eosin (H&E) staining (×200) and Masson staining (×200). Red arrows represent the corresponding pathological changes. **(B)** Serum level of ALT and AST were measured (*n* = 6). **(C)** Western blot analysis of α-SMA and Col. I with β-actin as the loading control. The assays were performed at least three times with similar results. Data are shown as the mean ± SD (*n* = 3) of one representative experiment. ^∗^*p* < 0.05, ^∗∗^*p* < 0.01 versus normal groups.

Intraperitoneal injection of CCl_4_ is a traditional method for inducing liver fibrosis. Mice treated with CCl4 were characterized by steatosis, expansion of cytoplasm, gradual conversion to myofibroblasts and inflammatory infiltration. The change of liver fibrosis tissues was detected by H&E staining and Masson staining. Histopathological analysis results showed that CCl_4_ treatment resulted in serious hepatic steatosis, necrosis, and formation of regenerative nodules and fibrotic septa compared to normal groups (**Figure 1A**). Additionally, serum AST and ALT activities were significantly increased in liver fibrosis model groups compared to normal groups (**Figure 1B**).

Next, the protein levels of α-SMA and Col. I were detected by using Western blot. These results indicated that the protein levels of α-SMA and Col. I were up-regulated in model groups compared with the control groups (**Figure 1C** and Supplementary Figure S2). Generally, the results demonstrated that the liver fibrosis models were established successfully.

### The Expression of TRAIL in Liver Fibrosis Tissues and TGF-β1-Induced HSCs

**FIGURE 2:**
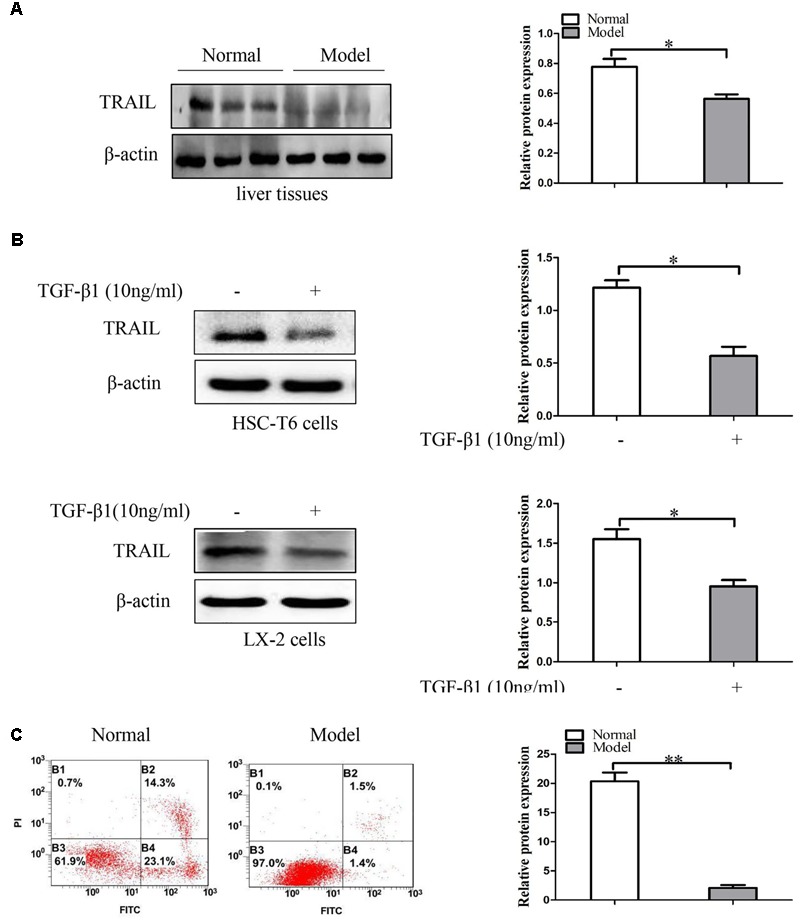
The expression of TRAIL in liver fibrosis tissues and TGF-β1-treated HSCs. **(A)** Western blot analysis and quantitative data for TRAIL expression in liver fibrosis tissues, β-actin as the loading control. **(B)** Western blot analysis and quantitative data show TRAIL expression in HSC-T6 cells and LX-2 cells treated with TGF-β1 (10 ng/ml) for 24 h. **(C)** The apoptotic rate of primary HSCs were determine by flow cytometry. The assays were performed at least three times with similar results. Data are shown as the mean ± SD (*n* = 3) of one representative experiment. ^∗^*p* < 0.05, ^∗∗^*p* < 0.01 versus normal groups.

In order to confirm the relationship between TRAIL and liver fibrosis, we evaluate the expression of TRAIL by using Western blot in liver fibrosis tissues. Analysis of Western blot displayed that the protein expression of TRAIL was decreased in model groups compared to the normal groups (**Figure 2A** and Supplementary Figures S2, S3A). Then HSC-T6 cells and LX-2 cells were stimulated with TGF-β1 (10 ng/ml) for 24 h, and then measured the protein levels of TRAIL. The results are in agreement with the above that the protein level of TRAIL was inhibited in HSCs treated with TGF-β1 (**Figure 2B**). Thus, down-regulation of TRAIL may be associated with the liver fibrosis.

In the following experiments, apoptosis in primary HSCs was quantified by using annexin V-FITC/PI double staining flow cytometry. The analysis of apoptosis showed that the apoptotic rate of primary HSCs was down-regulated in model groups compared to normal groups (**Figure 2C**). Collectively, these data indicate that TRAIL may be associated with the apoptosis of activated HSCs.

### Effect of miR-145 on Liver Fibrosis

#### The Expression of miR-145 *in Vivo* and *in Vitro*

**FIGURE 3:**
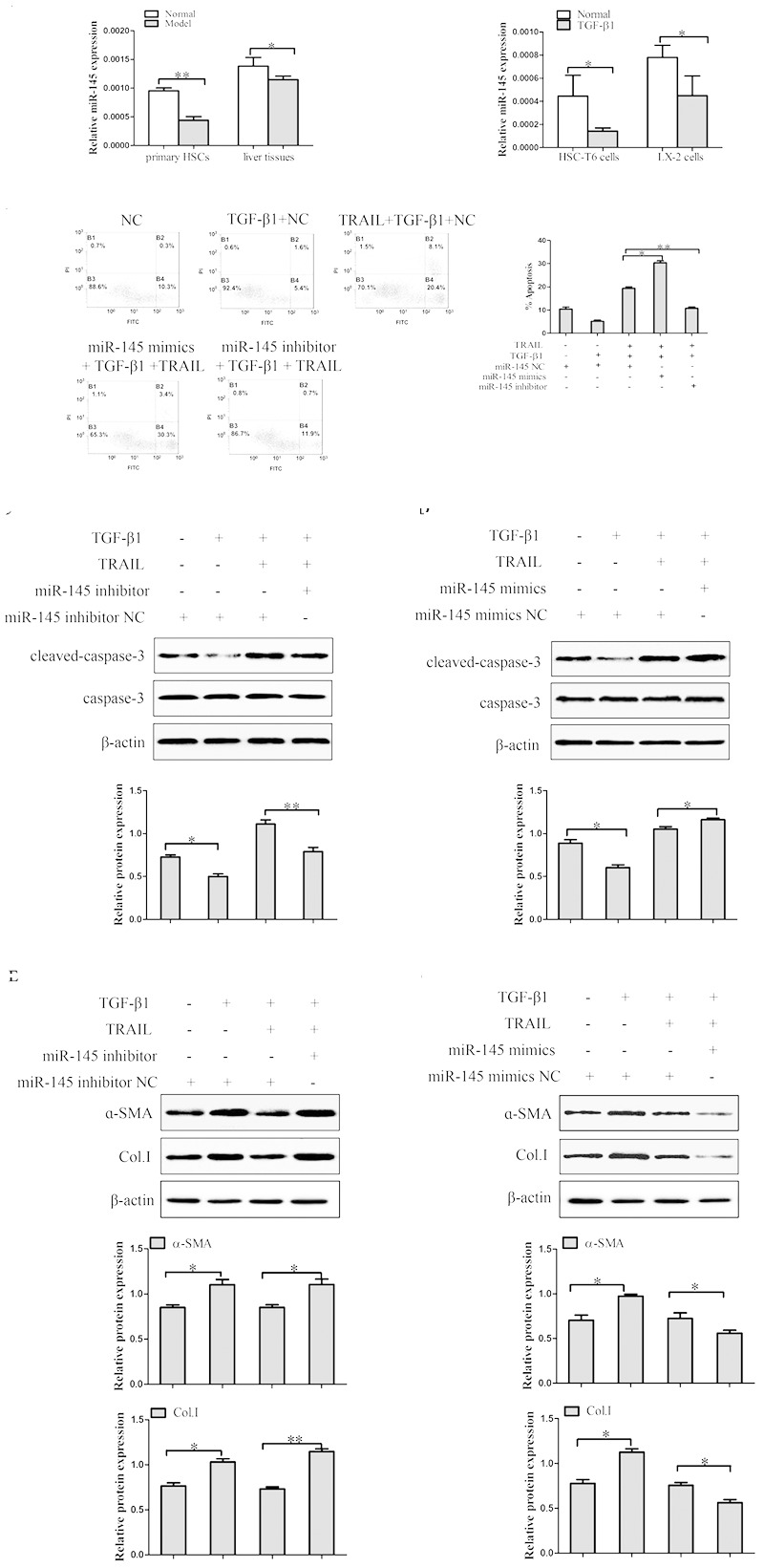
The effect of miR-145 on the apoptosis of TGF-β1-treated LX-2 cells induced by TRAIL. **(A)** The expression of miR-145 was detected by quantitative real-time PCR in liver fibrosis tissues, primary HSCs and activated HSCs. **(B)** miR-145 inhibitor and miR-145 mimics, respectively decreased and increased the rate of apoptosis in activated LX-2 cells that were pretreated with TRAIL (10 ng/ml) for 24 h. **(C)** The protein expression of cleaved of caspase-3 was measured by Western blot in activated LX-2 cells transfected with miR-145 mimics that were pretreated with TRAIL (10 ng/ml) for 24 h. **(D)** The protein expression of cleaved of caspase-3 was assessed by Western blot in activated LX-2 cells transfected with miR-145 inhibitor that were pretreated with TRAIL (10 ng/ml) for 24 h. **(E,F)** The expression of α-SMA and Col. I protein was determined by Western blot in activated LX-2 cells that were pretreated with TRAIL (10 ng/ml) for 24 h. The assays were performed at least three times with similar results. Data are shown as the mean ± SD (*n* = 3) of one representative experiment. ^∗^*p* < 0.05, ^∗∗^*p* < 0.01 versus normal groups.

In order to confirm the expression level of miR-145 in liver fibrosis tissues, primary HSCs and TGF-β1-induced HSCs. Results from quantitative real-time PCR indicated that the expression level of miR-145 was significantly lower in model groups compared to normal groups. In addition, we treated HSC-T6 cells and LX-2 cells with TGF-β1 (10 ng/ml) for 24 h. The results showed that the expression level of miR-145 was down-regulated in HSC-T6 cells and LX-2 cells treated with TGF-β1 compared to normal groups (**Figure 3A**).

#### Effect of miR-145 on TRAIL-Induced Apoptosis of LX-2 Cells

To investigate the related mechanism of miR-145 on the TRAIL-induced apoptosis of LX-2 cells, it is well established that the expression level of miR-145 is normally down-expression or over-expression by using miR-145 inhibitor and miR-145 mimics in LX-2 cells treated with TGF-β1 (10 ng/ml) and TRAIL (10 ng/ml). TRAIL could increase the rate of apoptosis and the protein level of cleaved caspase-3 in activated LX-2 cells. Interestingly, silencing of miR-145 remarkably decreased the apoptotic rate and the protein level of cleaved caspase-3 in LX-2 cells treatment with TRAIL (**Figures 3B,C**). More importantly, results showed that over-expression of miR-145 led to high apoptotic rate and increased the protein level of cleaved caspase-3 in LX-2 cells treatment with TRAIL (**Figures 3B,D**).

Because modulation of apoptosis of activated HSCs could be an important complementary pathway in preventing liver fibrosis, we examined whether the expression level of miR-145 was linked to the severity of liver fibrosis. As shown in **Figure 3E**, the miR-145 inhibitor increased the levels of α-SMA and Col. I protein compared to normal cells without inhibitor transfection in TRAIL-induced activated LX-2 cells. Whereas, over-expression of miR-145 reduced the protein levels of α-SMA and Col. I (**Figure 3F**). In the following experiment, we found that the over-expression of miR-145 could not affect the apoptosis in LX-2 cells without TRAIL treatment (Supplementary Figure S1). In general, the finding revealed that miR-145 could promote TRAIL-induced LX-2 cells apoptosis.

### Effect of miR-145 on the Expression of ZEB2 in LX-2 Cells

**FIGURE 4:**
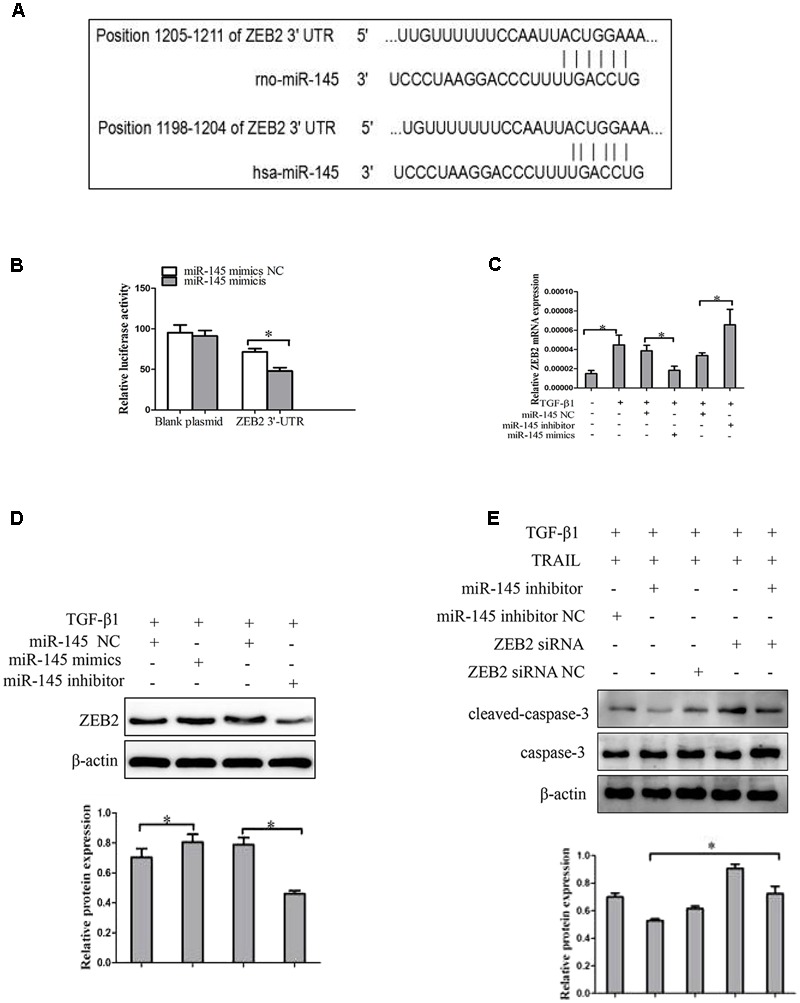
ZEB2 is a direct target of miR-145 in activated HSCs cells. **(A)** The target genes of miR-145 were predicted by using bioinformatics tools (TargetScan, CLIP-Seq, miRDB, and miRanda). **(B)** A miR-145 mimic or negative control and a luciferase vector encoding the wild-type or mutant ZEB2 3′-UTR region were co-transfected into LX-2 cells, and the relative luciferase activity was measured. **(C)** Quantitative real-time PCR analysis for mRNA expression of ZEB2 after transfected with miR-145 mimics, miR-145 inhibitor, or miR-145 NC. **(D)** Western blot analysis for protein expression of ZEB2 after transfected with miR-145 mimics, miR-145 inhibitor, or miR-145 NC. **(E)** Protein level of the cleaved of caspase-3 was determined by Western blot analysis in activated LX-2 cells co-transfected with miR-145 inhibitor and ZEB2-siRNA, The assays were performed at least three times with similar results. Data are shown as the mean ± SD (*n* = 3) of one representative experiment. ^∗^*p* < 0.05 versus normal groups.

Then we probed the potential mechanism by which miR-145 raises the apoptotic rate of activated LX-2 cells by TRAIL treatment. We used bioinformatics tools (TargetScan, CLIP-Seq, miRDB, and miRanda) to predict the miR-145 target genes. The miRNA target prediction algorithm predicted that the 3′-UTR of ZEB2 mRNA contains putative miR-145 binding sites (**Figure 4A**). ZEB2 has been confirmed as miR-145 target gene in cancer cells. To further verify whether ZEB2 is a target of miR-145, we evaluate ZEB2 response to miR-145 by using double luciferase reporter assay. Results showed the 3′-UTR conveyed decreased expression of ZEB2 (**Figure 4B**). At the same time, the results from qRT-PCR showed that the ZEB2 mRNA level was significantly lower in cells transfected with miR-145 mimics than in the cells in the negative control group, whereas transfection with miR-145 inhibitor increased the levels of ZEB2 mRNA in cells (**Figure 4C**). The inverse correlation between miR-145 and ZEB2 mRNA expression was further confirmed by Western blot analysis (**Figure 4D**). Next, we further measured whether miR-145 enhanced TRAIL-induced apoptosis of LX-2 cells by targeting ZEB2, the protein expression of cleaved caspase-3 were detected by using Western blot in LX-2 cells that was co-transfection of miR-145 inhibitor and ZEB2-siRNA, compared with miR-145 inhibitor. We found that the protein level of cleaved caspase-3 was increased (**Figure 4E**). Taken together, these results provided evidence that miR-145 specifically targets the 3′-UTR regions of ZEB2 and thus inhibits the expression of ZEB2.

### The Expression of ZEB2 in Liver Fibrosis Tissues and TGF-β1-Induced HSCs

**FIGURE 5:**
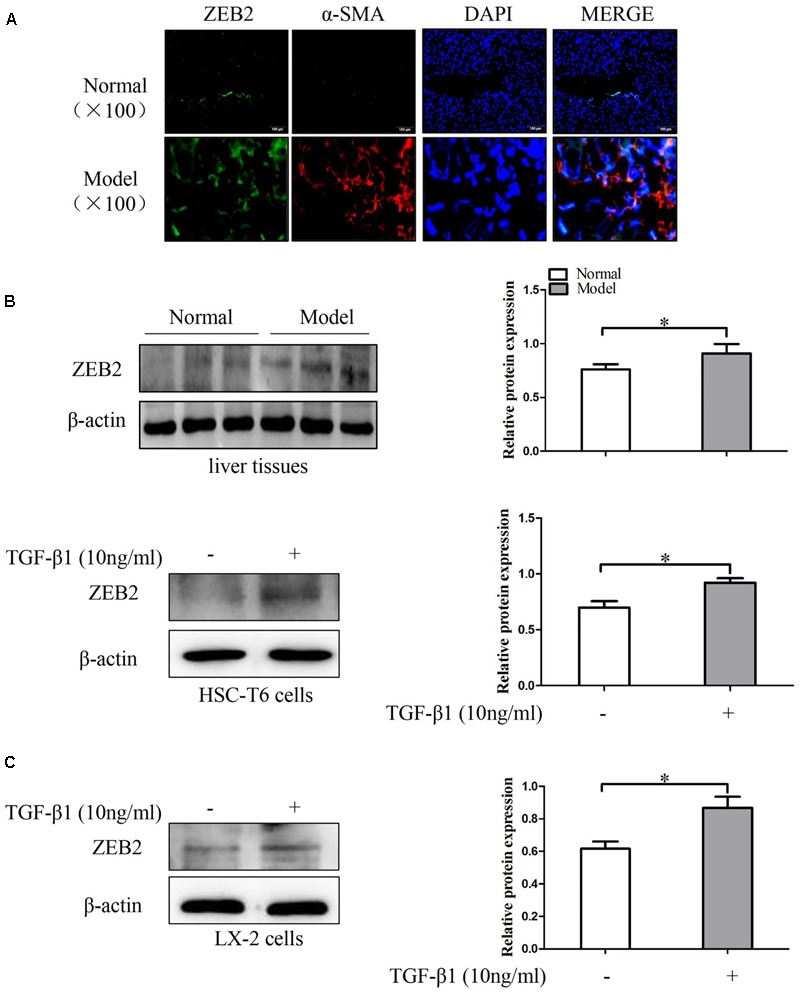
The expression of ZEB2 in mice liver fibrosis tissues and TGF-β1-treated HSCs. **(A)** Immunofluorescence staining with ZEB2 and α-SMA were performed to detect the co-localization of ZEB2 (red) and α-SMA (green) in liver fibrosis tissues. Representative images of Immunofluorescence staining are presented (×100). **(B)** The level of ZEB2 was evaluated by Western blot analysis in liver fibrosis tissues. **(C)** HSC-T6 cells and LX-2 cells were treated with TGF-β1 (10 ng/ml) for 24 h, and then protein expression of ZEB2 were determine by Western blot analysis. The assays were performed at least three times with similar results. Data are shown as the mean ± SD (*n* = 3) of one representative experiment. ^∗^*p* < 0.05 versus normal groups.

To investigate the expression patterns of ZEB2 in liver fibrosis tissues and activated HSCs, the co-labeling ZEB2 and α-SMA was performed for colocalization. As immunofluorescence results shown, there were abundant ZEB2 labeling in the liver fibrosis tissues. Importantly, the double immunofluorescence staining showed that the expression of ZEB2 was primarily co-localized with α-SMA, indicating that HSCs can be considered as one of the main sources of the ZEB2 levels present in liver fibrosis tissues (**Figure 5A** and Supplementary Figures S2, S3B). Correspondingly, we detected the expression of ZEB2 in liver fibrosis tissues. Results from Western blot showed that the expression of ZEB2 was significantly increased in model groups compared to normal groups (**Figure 5B**). To elucidate whether TGF-β1 influence the expression of ZEB2, the HSC-T6 cells and LX-2 cells were treated with TGF-β1 (10 ng/ml) for 24 h. Interestingly, TGF-β1 could simultaneously enhance the expression of ZEB2 in HSCs (**Figure 5C**). These data supported our observation that ZEB2 may be associated with liver fibrosis.

### Effect of ZEB2 on TRAIL-Induced Apoptosis in LX-2 Cells

**FIGURE 6:**
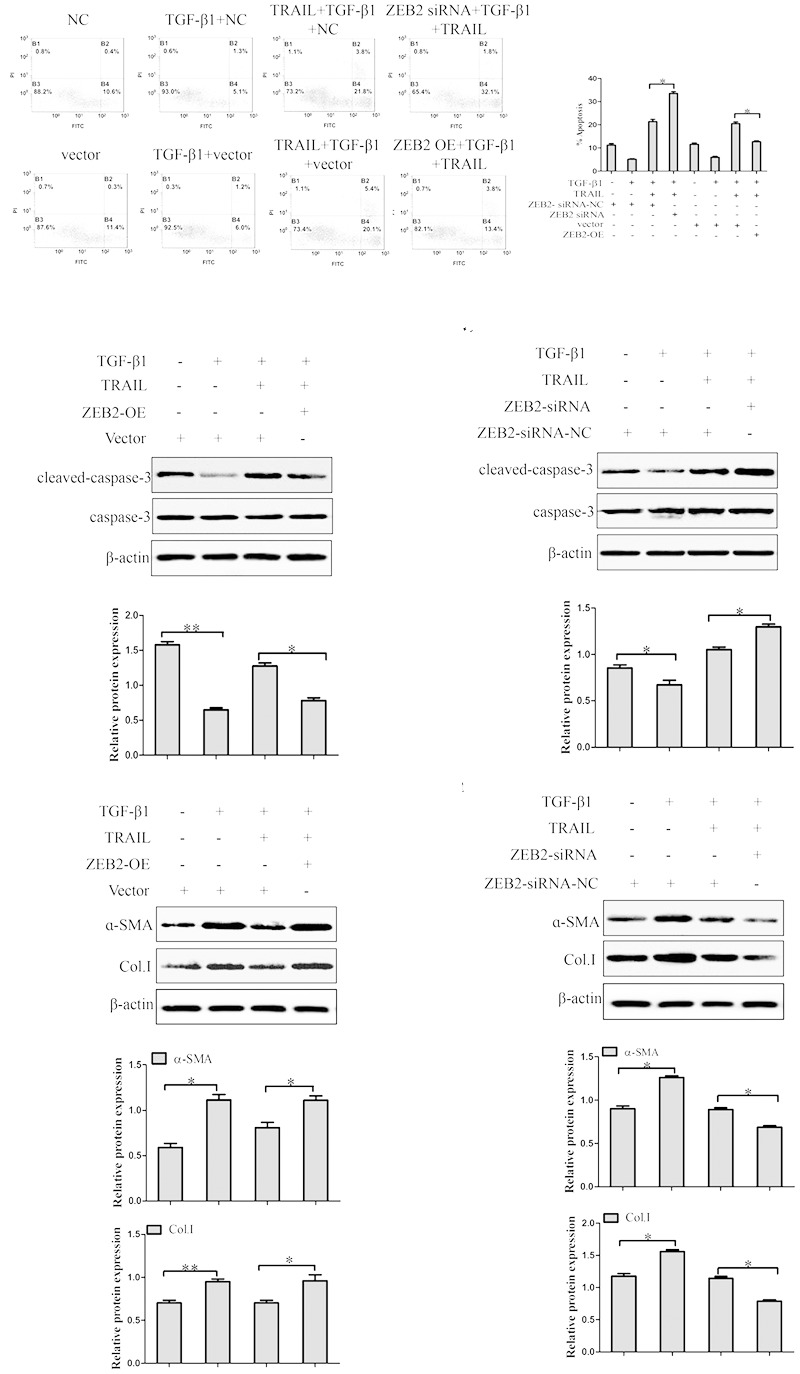
The effect of ZEB2 on TRAIL-induced apoptosis of LX-2 cells. **(A)** The LX-2 cells were transfected with ZEB2-siRNA and ZEB2-OE, respectively that were pretreated with TRAIL (10 ng/ml) for 24 h, and then apoptotic rate of LX-2 cells were measured by flow cytometry. **(B)** The LX-2 cells were transfected with ZEB2-OE that were pretreated with TRAIL (10 ng/ml) for 24 h, and then protein level of cleaved of caspase-3 was measured by Western blot. **(C)** The LX-2 cells were transfected with ZEB2-siRNA, and then protein level of cleaved of caspase-3 was measured by Western blot analysis. **(D,E)** The LX-2 cells were transfected with ZEB2-siRNA and ZEB2-OE, respectively, and then level of α-SMA and Col. I was detected by Western blot analysis. The assays were performed at least three times with similar results. Data are shown as the mean ± SD (*n* = 3) of one representative experiment. ^∗^*p* < 0.05, ^∗∗^*p* < 0.01 versus normal groups.

In order to further investigate the role of ZEB2 in TRAIL-induced apoptosis of activated LX-2 cells. The LX-2 cells were transfected with ZEB2 siRNA or ZEB2 plasmid. Apoptosis assays results by using the annexin V-FITC/PI double staining showed that TRAIL could obviously increase the rate of apoptosis and the level of cleaved caspase-3 in activated LX-2 cells. However, the effect of TRAIL on the apoptotic rate and level of cleaved caspase-3 could be partially rescued by the over-expression of ZEB2 (**Figures 6A,B**). The down-regulation of ZEB2 enhanced the TRAIL-induced apoptosis of LX-2 cells and the level of cleaved caspase-3 (**Figures 6A,C**). Then we further examined the expression of α-SMA and Col. I at protein levels. Results of Western blot showed that ZEB2 plasmid resulted in increased the protein levels of α-SMA and Col. I (**Figure 6D**). Whereas, the protein levels of α-SMA and Col. I were markedly decreased by ZEB2 siRNA treatment (**Figure 6E**). In general, our data indicated that ZEB2 inhibits TRAIL-induced apoptosis of activated LX-2 cell.

### Effect of ZEB2 on NF-κB Activity in TRAIL-Induced Apoptosis of LX-2 Cells

**FIGURE 7:**
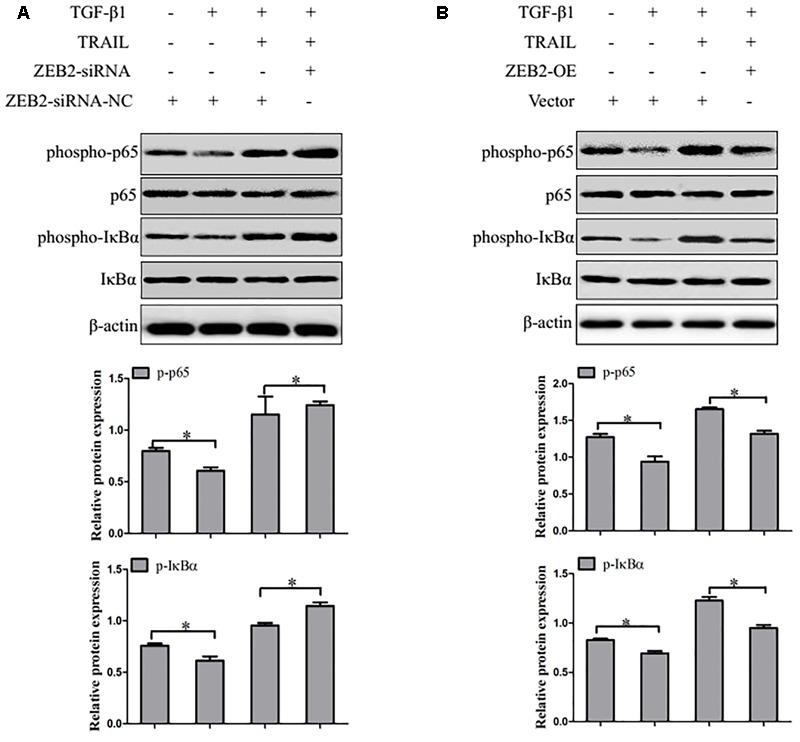
ZEB2 negatively regulated the activity of NF-κB. **(A)** The LX-2 cells transfected with ZEB2-OE, and then protein expression of the phospho-p65 and phospho-IκBα was determined by Western blot analysis. **(B)** The LX-2 cells transfected wit ZEB2-siRNA, and then protein expression of the phospho-p65 and phospho-IκBα was determined by Western blot analysis. The assays were performed at least three times with similar results. Data are shown as the mean ± SD (*n* = 3) of one representative experiment. ^∗^*p* < 0.05 versus normal groups.

Recent evidence suggested that activation of the transcription factor NF-κB plays a critical role in HSCs apoptosis. Therefore, we detected whether ZEB2 regulates NF-κB signaling activity in TRAIL-induced apoptosis of activated LX-2 cells. LX-2 cells were treated with ZEB2 siRNA or ZEB2 plasmid. Western blot analysis found that the protein levels of the phospho-IκBα and phospho-p65 were significantly increased after ZEB2-siRNA transfection in TRAIL-induced activated LX-2 cells (**Figure 7A**). In contrast, the protein levels of the phospho-IκBα and phospho-p65 were decreased following ZEB2 over-expression (**Figure 7B**). These results suggested that the effects of ZEB2 might be mediated by NF-κB signaling pathway in TRAIL-induced apoptosis of LX-2 cells.

## Discussion

In this study, we initially compared the expression levels of miR-145 and TRAIL in liver fibrosis tissues and activated HSCs. We found that miR-145 and TRAIL was significantly down-regulated in liver fibrosis tissues and activated HSCs. Our results further showed that over-expression of miR-145 or knockdown of ZEB2 enhanced the apoptosis of activated HSCs induced by TRAIL. Next, we demonstrated that miR-145 regulated the apoptosis of activated HSCs induced by TRAIL by targeting ZEB2 through NF-κB signaling pathway. Therefore, we deduced that the up-regulation of miR-145 contributed to the apoptosis of TRAIL-induced activated HSCs.

Upon the liver injury, quiescent HSCs become activated leading to enhance the expression of α-SMA and production of ECM (Sato et al., 2003; Higashi et al., 2017). However, the reversibility of liver fibrogenesis has been accepted and is an underlying strategy for the resolution of liver fibrosis. Applications of miRNA have been frequently reported in a variety of fibrotic diseases. Gene therapies based on miRNAs have been proposed as a new therapeutic approach for fibrotic diseases (Jiang et al., 2016). miRNA has been studied to contribute or attenuate the pathophysiology of liver fibrosis, and several miRNA have been reported to regulate activation of HSCs (Noetel et al., 2012). Now, evidence has emerged that miR-145 was significantly lower in cardiac fibrosis and keloid fibroblasts. Importantly, miR-145 is also described as an anti-proliferative gene in HSCs during fibrogenesis (Zhou et al., 2016; Wei et al., 2017). The aim of the present study was to confirm whether miR-145 had a regulatory effect on HSC apoptosis.

TRAIL, belongs to the TNF family, selectively induces tumor cell apoptosis and has a little impact on most normal cells. The members of this family, include TRAIL and its five related receptors, are expressed and serve as pro-apoptotic regulators in most malignant gynecological tumor cells (Merino et al., 2007; Johnstone et al., 2008). Researchers showed that TRAIL-induced apoptosis of activated human HSCs might act as a valuable new model for studying human liver fibrosis (Xu et al., 2005). Recently, it has been reported that TRAIL plays a vital role in the recovery of liver fibrosis (Park et al., 2009). In the present study, we have identified that miR-145 promotes the apoptosis of activated LX-2 cells induced by TRAIL during liver fibrosis.

Our data indicated that the protein level of TRAIL and the apoptotic rate of activated HSCs were decreased during liver fibrosis, while the expression level of miR-145 remains lower. In addition, we measured that the effect of miR-145 on apoptosis of LX-2 cells induced by TRAIL *in vitro* by transfecting with miR-145 inhibitor or miR-145 mimics, suggesting that while miR-145 was suppressed, the apoptotic rate of TRAIL-induced activated LX-2 cells and level of cleaved caspase-3 were down-regulated compared to LX-2 cells treated with mimics and normal cells. However, the over-expression of miR-145 resulted in enhanced TRAIL-induced LX-2 cells apoptosis and the level of cleaved caspase-3. Interestingly, the over-expression of miR-145 by transfecting with miR-145 mimics followed by treatment with TRAIL led to a down-regulated in the protein levels of α-SMA and Col. I in TRAIL-induced apoptosis of LX-2 cells. In contrast, the knockdown of miR-145 increased the protein levels of α-SMA and Col. I. Based on the experimental data discussed above, we conclude that miR-145 enhanced the apoptotic rate of TRAIL-induced activated LX-2 cells.

The mechanisms of miR-145 in positively regulating apoptosis induced by TRAIL and down-regulate TGF-β1-mediated ECM production remain unknown. We also indicated an up-regulation in the expression level of ZEB2 in activated LX-2 cells compared to without activated LX-2 cells. ZEB2, a zinc-finger E-box binding proteins, has been considered as an important inhibitor of apoptosis and a useful therapeutic candidate. Additionally, NF-κB-mediated transcriptional activation is known as a central pathophysiological mechanism in the process of HSCs activation (Sun and Karin, 2008).

Previous studies showed that active NF-κB plays a pivotal role in preventing apoptosis of activated HSCs (Vasiliou et al., 2000). It was well reported the inhibition of NF-κB signaling pathway is usually associated with the induction of apoptosis in activated HSCs and the resolution of experimentally induced liver fibrosis (Friedman, 2010). For example, resveratrol can induce HSCs apoptosis through the inhibition of NF-κB signaling pathway and changed regulation of NF-κB-dependent gene transcription *in vitro* and *in vivo* (Habens et al., 2005; Chavez et al., 2008). Notably, many studies showed that ZEB2 is highly expressed in mast cells, which is connected with NF-κB signaling pathway (Barbu et al., 2012). In our present study, we found that miR-145 was negatively regulated expression of ZEB2 during the stage of apoptosis of TRAIL-induced activated LX-2 cells. ZEB2-siRNA treatment significantly increased the phospho-IκBα and phospho-p65 expression, suggesting that ZEB2 negatively regulates NF-κB signaling activity. Therefore, miR-145 might down-regulated ZEB2 through NF-κB signaling pathway, which enhanced sensitivity of LX-2 cells to TRAIL-induced apoptosis and subsequent protection against TGF-β1-induced liver fibrosis. In general, the specific regulatory mechanism by which miR-145 promotes apoptosis of LX-2 cells induced by TRAIL is still to be determined future.

In summary, we provided novel insights into the mechanism by which miR-145 plays a protective role against TGF-β1-induced fibrogenesis. Our results indicated that compared to normal liver tissues and quiescent HSCs, liver fibrosis tissues and activated HSCs showed low expression of miR-145. In addition, miR-145 could function as a regulator against fibrogenesis by targeting ZEB2 through NF-κB signaling pathway. Theoretically, it seems that medicines targeting miR-145 can appear as a potential strategy to against liver fibrosis.

## Author Contributions

LZ and XW designed the experiments and took part in the critical revision of the manuscript. JY carried out experiments and participated in drafting of the manuscript. CH provided a series of experimental instructions and help. XM and XL conducted the primary screening test of relevant drugs. TX and LP analyzed the experimental results. CL and DZ analyzed and interpreted the data. QL and SC assisted with experiments on animals.

## Conflict of Interest Statement

The authors declare that the research was conducted in the absence of any commercial or financial relationships that could be construed as a potential conflict of interest. The reviewer ZL and handling editor declared their shared affiliation.
