# Supplementary figures and images for: MicroRNA-145 Increases the Apoptosis of Activated Hepatic Stellate Cells Induced by TRAIL through NF-κB Signaling Pathway
Source: Front Pharmacol. 2018 Jan 12;8:980. doi: 10.3389/fphar.2017.00980 (PMC5770373; doi:10.3389/fphar.2017.00980)

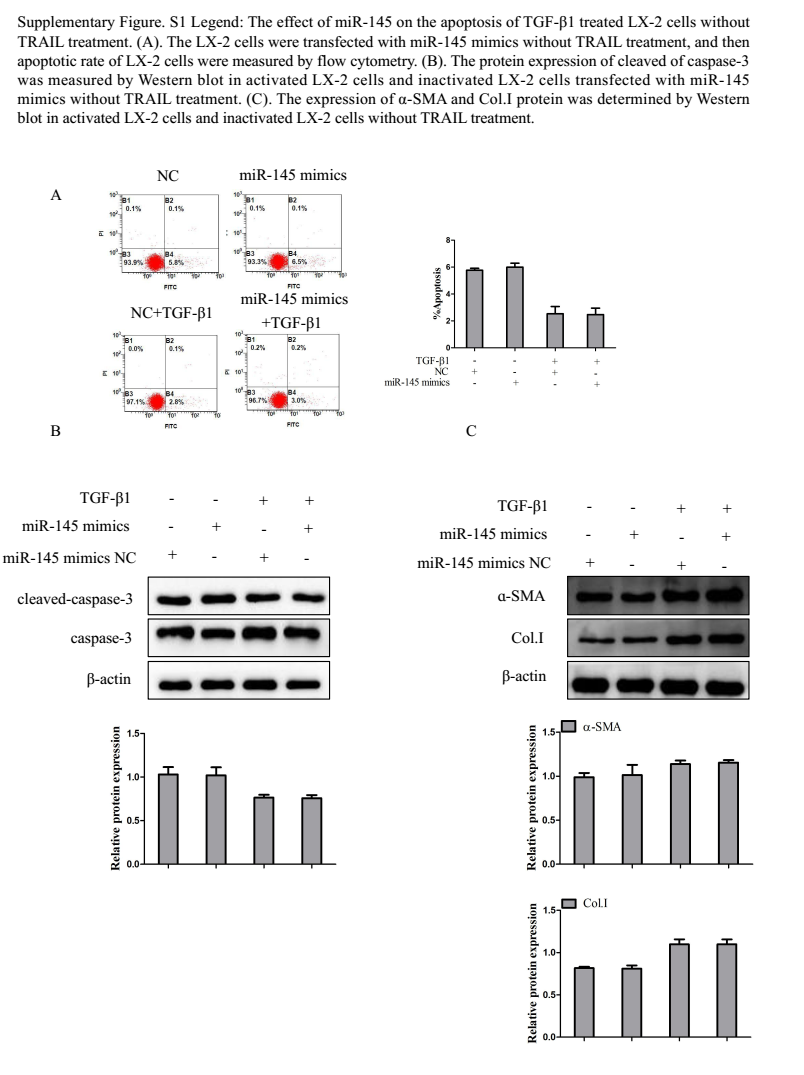

Supplement: Supplementary file 1 [file Image_1.TIF]
